# DOPAL derived alpha-synuclein oligomers impair synaptic vesicles physiological function

**DOI:** 10.1038/srep40699

**Published:** 2017-01-13

**Authors:** N. Plotegher, G. Berti, E. Ferrari, I. Tessari, M. Zanetti, L. Lunelli, E. Greggio, M. Bisaglia, M. Veronesi, S. Girotto, M. Dalla Serra, C. Perego, L. Casella, L. Bubacco

**Affiliations:** 1 Department of Biology, University of Padova, Italy; 2 Department of Chemistry, University of Pavia, Italy; 3 Institute of Biomedical Technologies, National Research Council of Italy, Segrate (Milan), Italy; 4 Institute of Biophysics, National Research Council of Italy, Trento, Italy; 5 Laboratory of Biomolecular Sequence and Structure Analysis for Health, Bruno Kessler Foundation, Trento, Italy; 6 Department of Drug Discovery and Development, Istituto Italiano di Tecnologia, Genova, Italy; 7 Department of Pharmacological and Biomolecular Sciences, University of Milan, Italy

## Abstract

Parkinson’s disease is a neurodegenerative disorder characterized by the death of dopaminergic neurons and by accumulation of alpha-synuclein (aS) aggregates in the surviving neurons. The dopamine catabolite 3,4-dihydroxyphenylacetaldehyde (DOPAL) is a highly reactive and toxic molecule that leads to aS oligomerization by covalent modifications to lysine residues. Here we show that DOPAL-induced aS oligomer formation in neurons is associated with damage of synaptic vesicles, and with alterations in the synaptic vesicles pools. To investigate the molecular mechanism that leads to synaptic impairment, we first aimed to characterize the biochemical and biophysical properties of the aS-DOPAL oligomers; heterogeneous ensembles of macromolecules able to permeabilise cholesterol-containing lipid membranes. aS-DOPAL oligomers can induce dopamine leak in an *in vitro* model of synaptic vesicles and in cellular models. The dopamine released, after conversion to DOPAL in the cytoplasm, could trigger a noxious cycle that further fuels the formation of aS-DOPAL oligomers, inducing neurodegeneration.

Parkinson’s disease (PD) is a neurodegenerative disorder characterized by the prominent and progressive loss of dopaminergic neurons in the *substantia nigra pars compacta (SNpc*)^1^. Loss of these neurons suggests that this type of cells is particularly vulnerable to the pathological mechanisms leading to the disease. Based on this assumption, potential toxicity mechanisms associated with the presence of dopamine (DA) or DA catabolites in neurons, have been extensively investigated in the past^2^,^3^,^4^. In this context, the role of 3,4-dihydroxyphenylacetaldehyde (DOPAL) is of particular interest^5^. This catabolite is generated from DA by monoamine oxidase (MAO) and is highly reactive and toxic. It presents an aldehyde moiety that drives DOPAL to react with the amino groups of proteins, and a catechol moiety, which can be oxidized to quinone and lead to polymerization. In healthy neurons, DOPAL is effectively detoxified by aldehyde dehydrogenase (ALDH), which converts DOPAL to 3,4-dihydroxyphenylacetic acid (DOPAC). Interestingly, DOPAL:DA ratios were found to be higher in PD brains than the corresponding ratios in control subjects^6^, and the cytosolic ALDH gene was found to be down regulated in PD brains^7^. Cytosolic ALDH is also differentially expressed among dopaminergic neurons, and the subpopulation of DA neurons which lack the enzyme is more vulnerable to neurodegeneration^8^. Exposure to ALDH inhibitors^9^, such as the fungicide benomyl^10^,^11^ or disulfiram^12^, causes DOPAL accumulation and have been associated with an increased risk to develop PD; ALDH-KO mice show a Parkinsonian phenotype, DA neuronal death and DOPAL accumulation^13^.

Another key player in the working hypothesis of this study is the presynaptic protein alpha-synuclein (aS). aS is the main constituent of Lewy bodies (LBs), which are inclusions composed of proteins and lipids found in PD brains^14^. Mutations in the aS gene and gene multiplications are associated to inherited PD forms^15^. The physiological function of aS has been associated with neurotransmitter release^16^, synaptic vesicle trafficking^17^ and SNARE complex assembly^18^, but the details of its functions are yet to be elucidated. However, aS knock-out mice are viable and present only small alterations in DA level and release^19^.

One relevant property of aS is its propensity to aggregate and to interact with DA^20^, leading to the formation of a heterogeneous ensemble of oligomers, which then convert to amyloid fibrils that are deposited in LBs. Different toxicity mechanisms, such as mitochondrial dysfunction^21^, autophagy impairment^22^ and membrane damage^23^, have been ascribed to the diverse forms of aS aggregates reported in the literature^24^. Therefore, it is still unclear which are the most relevant aggregates for the pathology, and what their primary toxic effect may be. The facts that aS (i) is highly concentrated at the presynaptic terminal, i.e. about 40 μM^25^, (ii) has 15 lysines (Lys) in its sequence of 140 amino acids and (iii) is natively unfolded^26^, suggest that aS may be a target for DOPAL among synaptic proteins^27^. Concurrently, it was shown that DOPAL can cause aS oligomerization *in vitro* and in cell models^4^,^28^,^29^,^30^,^31^. Herein, starting from the detrimental effect caused by DOPAL-induced aS oligomerization on the function of synaptic vesicles, we investigated the structural and functional features of aS-DOPAL oligomers. This analysis allowed us to propose a mechanism by which synaptic vesicles could be damaged. In particular, we show that the oligomers can permeabilise cholesterol-containing lipid membranes mimicking synaptic vesicles *in vitro*, suggesting that the synergistic effect of aS and DOPAL accumulation in DA neurons may lead to the formation of oligomers able to negatively impact on the structure and function of synaptic vesicles.

## Results

### DOPAL-modified aS can damage cellular vesicles and affect their mobility in BE(2)-M17

DOPAL has previously been shown to induce the formation of aS oligomers *in vitro*^4^,^28^,^29^,^30^,^31^. To assess whether the DOPAL-induced oligomerization of aS may cause synaptic vesicle damage, we used a pH-sensitive fluorescent protein directly targeted to synaptic vesicles (synaptobrevin2-pHluorin), as a reporter in a total internal reflection fluorescence microscopy (TIRFM) experiment^32^,^33^ in live cells. Synaptobrevin2-pHluorin fluorescence intensity increases if pH becomes basic, i.e. if vesicle fusion and/or permeabilisation occurs. Fluorescence intensity variation was measured in BE(2)-M17 cells overexpressing aS, and treated with 100 μM DOPAL, to monitor permeabilisation events. Normally, only vesicles fusing with the plasma membrane become fluorescent and then, after endocytosis, they are immediately switched off^32^. If DOPAL alters vesicles permeability, we expect an increase in the intensity, and in the persistence of the fluorescent signal associated with the vesicular structure. This vesicle-targeted fluorescent probe allowed also to study vesicles mobility in live cells.

**Figure 1:**
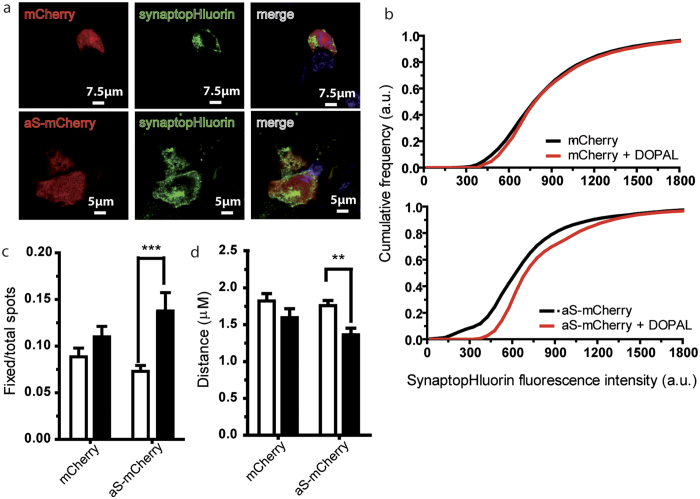
DOPAL effect on vesicle trafficking in aS overexpressing cells. (**a**) Confocal images of BE(2)-M17 cells transfected with mCherry or aS-mCherry and synapto-pHluorin. (**b**) Normalized fluorescence intensity/number of vesicles cumulative graphs for mCherry and aS. Black and red traces refer to untreated cells, and cells treated with DOPAL 100 μM for 24 hours. (**c**) Histograms express the ratio between fixed (see Methods for details) and total vesicles over a certain period of time, i.e. the number of vesicles showing a high fluorescent value for the whole time span of the measure, for mCherry or aS-mCherry overexpressing cells untreated or treated with 100 μM for 24 hours. (**d**) Average distance covered by vesicles in cells overexpressing mCherry or aS-mCherry at time 0 and after 100 μM DOPAL treatment at 24 hours. White and black stand for control cells and cells treated with 100 μM DOPAL for 24 hours. Bars represent mean ± SEM from n = 25–30 cells from at least three independent experiments. Asterisks indicate statistical significance by two-way ANOVA (**p < 0.01, ***p < 0.001).

Synaptobrevin2-pHluorin and mCherry or aS-mCherry were co-transfected in BE(2)-M17 neuroblastoma cells (Fig. 1a). TIRFM was used to monitor changes in vesicles fluorescence intensity and in their mobility within 150 nm from the plasma membrane. The variation in the cumulative distributions of the fluorescence intensity of the observed vesicles for cells overexpressing aS-mCherry (or mCherry as control) when untreated or upon 24 h treatment with 100 μM DOPAL was calculated (Fig. 1b). After 24 h of DOPAL treatment, the inflexion point of the curve reporting the cumulative distribution of the fluorescence intensity moves toward higher intensities values compared to time 0 in the aS-mCherry overexpressing cells, but not in control. This result suggests that DOPAL-modified aS or aS-DOPAL oligomers can cause a gradient driven efflux of H^+^ in vesicles that leads to an increase in pH, which is responsible for the increase in the fluorescence intensity. Consistently, the number of vesicles showing a high fluorescent value for the whole time span of the measure (i.e. the number of unchanged vesicles over a certain period of time) is increased in the aS overexpressing cells after DOPAL treatment (Fig. 1c).

Interestingly, there is also a significant reduction in the average distance covered by vesicles, in cells overexpressing aS-mCherry after the DOPAL treatment compared to the control (Fig. 1d). No difference is present between mCherry and aS-mCherry overexpressing cells before DOPAL treatment, suggesting that the effect we measured depends on the synergic action of aS and DOPAL, likely through DOPAL-induced modification and oligomerisation of aS.

### DOPAL affects synaptic vesicles pool in mice primary cortical neurons

**Figure 2:**
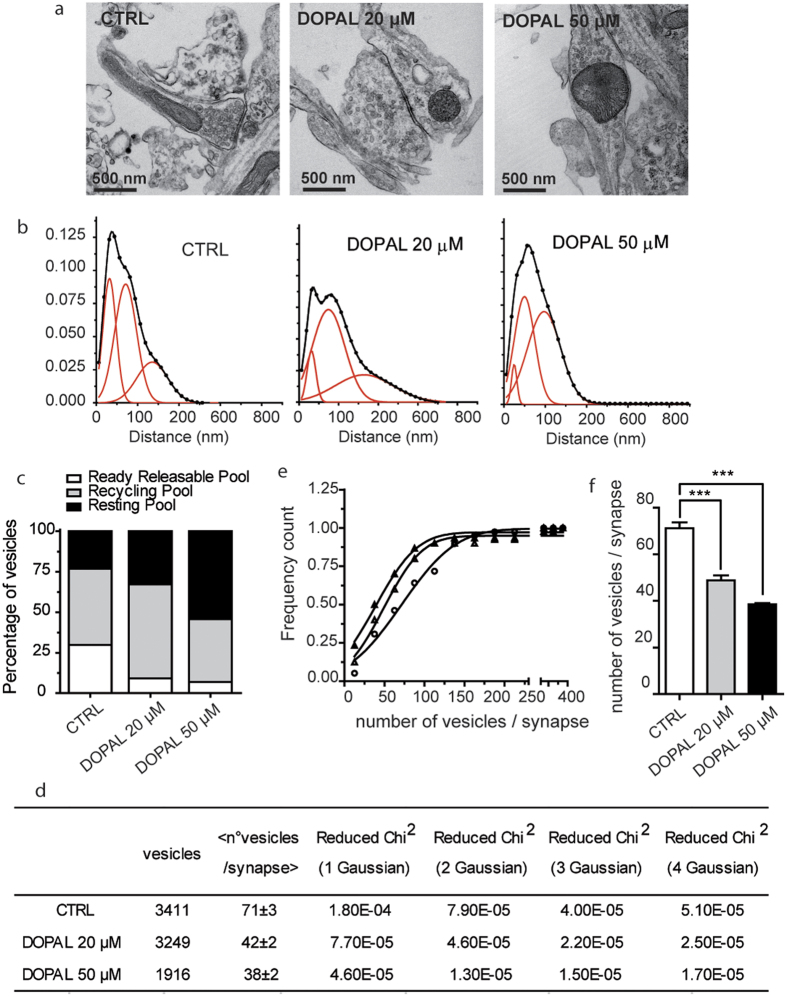
DOPAL effect on synaptic vesicles in primary mouse neurons. (**a**) TEM images of mice primary neurons synapses not treated (CTRL) and treated with 20 μM and 50 μM DOPAL. (**b**) Frequency distribution of vesicles distance from the active zone of primary neurons in control, treated with 20 μM and 50 μM DOPAL (from left to right). Data were fitted with a three Gaussian function (OriginPro8). The dots represent the experimental points, the black continuous line is the best fit generated by sum of the contributing Gaussian curves (red continuous lines). (**c**) Grouped stack column plot of the percentage of area under curve of the three vesicle populations, representing the percentage of vesicles belonging to the ready-releasable (white), recycling (grey) and resting (black) pools in control neurons and in neurons treated with 20 μM and 50 μM DOPAL. (**d**) Summary of the parameters derived from the TEM images analysis: number of total vesicles, number of total synapses, average number of vesicles per synapse, and the value of the reduced Chi-Squared for fits with one, two, three and four Gaussian curves (OriginPro8), suggesting that the best fit is the one with three Gaussian curves. (**e**) Cumulative distributions of the number of vesicles per synapse show that the number of vesicles per synapse is higher in controls (full triangle) than in neurons treated with 20 μM and 50 μM DOPAL (empty triangles and empty circles, respectively). (**f**) Histograms representing the average inflection point values of the cumulative distribution of the number of vesicles/synapse showing a significant reduction after 20 μM and 50 μM DOPAL treatment. Bars represent mean ± SEM from n = 30–39 synapses from at least three independent experiments. Asterisks indicate statistical significance by two-way ANOVA (***p < 0.001).

An independent approach was used to verify that synaptic vesicles’ functionality is affected by the synergic effect of aS and DOPAL. We used mice primary cortical neurons that express endogenous aS^25^ and treated them at DIV14 with DOPAL. After overnight treatment with 0 uM, 20 uM or 50 uM DOPAL, neurons were prepared for TEM imaging and synapses were imaged (Fig. 2a). The relative distributions of the distances between vesicles and the active zone of the synapses was measured as previously reported^34^ (Fig. 2b). From a quantitative comparison between the distributions corresponding to untreated and to 20 μM or 50 μM DOPAL treated neurons, DOPAL leads to a reduction in the readily releasable pool of synaptic vesicles, with an increase in the fraction of vesicles belonging to the resting pool (Fig. 2c). The counting of the individual vesicles in the TEM images also allowed analysis of the impact of DOPAL treatment and of the aS-DOPAL molecules on the average number of vesicles per synapse. The inflection point of the cumulative distribution of the number of vesicles per synapses is ~71 ± 3 for the untreated neurons and drops to 49 ± 2 and 38 ± 2 for the 20 μM and 50 μM DOPAL treated neurons, respectively, and it is decreasing in a [DOPAL]-dependent way (Fig. 2e,f).

### aS-DOPAL oligomers are formed in BE(2)-M17, HEK293 cells and primary cortical neurons

**Figure 3:**
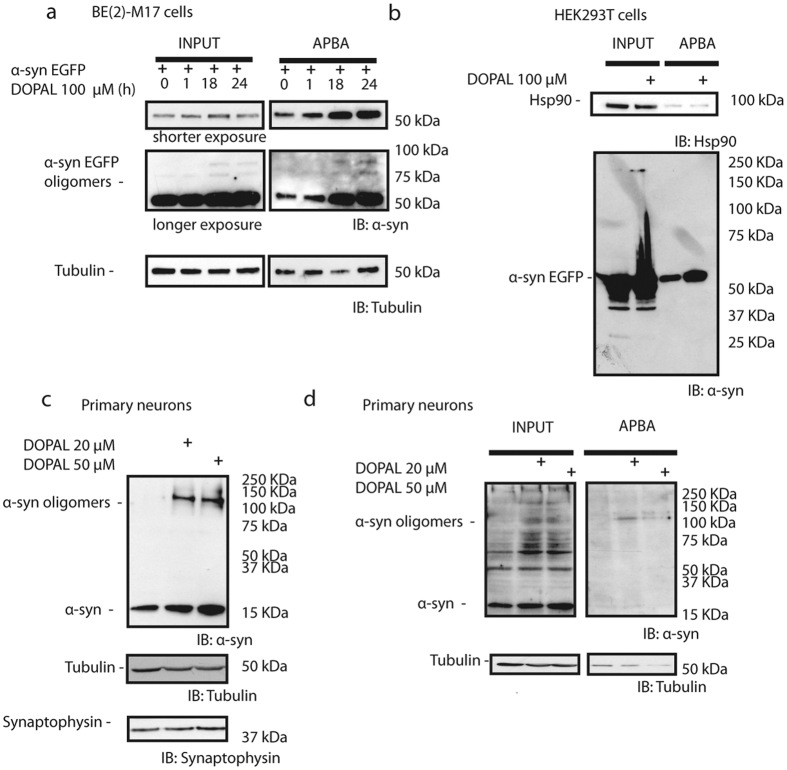
aS-DOPAL oligomers are found in neuronal cell models upon DOPAL treatment. (**a**) Western blot analysis of BE(2)-M17 cells overexpressing aS-mCherry and treated with 100 μM DOPAL for 1 hour, 18 hours or 24 hours. aS antibody detected aS monomers and oligomers (left) and after the pull-down with ABPA resin (right) the band showing monomeric aS modified by DOPAL is larger than the control after 18 or 24 hours treatment. (**b**) Western blot analysis of HEK293 cells treated with 100 μM DOPAL showing DOPAL-induced aS oligomers at high molecular weight (left) and the pull-down with ABPA resin (right) show that DOPAL-modified aS monomer is accumulated upon treatment. (**c**) Western blot of primary cortical neurons upon 20 μM and 50 μM DOPAL treatment show monomeric aS and aS-DOPAL oligomers accumulation. (**d**) ABPA resin pull-down from neuronal lysate of control and treated samples suggested that the accumulated monomeric aS is modified by DOPAL.

To verify whether previously reported synaptic vesicles dysfunction in BE(2)-M17 and primary neurons could be associated with the presence of aS-DOPAL oligomers, BE(2)-M17 cells overexpressing aS tagged with mCherry (aS-mCherry) were treated with 100 μM DOPAL. After 1, 18 or 24 h, cell lysates were subjected to the aminophenylboronic acid (APBA) resin, which allowed the pull-down of DOPAL-modified proteins^8^. Both total cell lysates and the pull-down samples were analysed by western blot using an antibody against aS (Fig. 3a). Total cell lysate lanes show bands corresponding to oligomeric species when cells were treated with DOPAL, and the correspondent ABPA pull-down lanes show an increase in the detected aS, likely modified by DOPAL. These results suggest that aS monomers were covalently modified by DOPAL molecules, and formed oligomers upon DOPAL treatment. The formation of aS-DOPAL oligomers was not detected in an alternative cell line (HEK293T) overexpressing aS fused with the fluorescent protein EGFP and treated with DOPAL (Fig. 3b) in spite of the presence of oligomeric species in the input.

Interestingly, the formation of aS-DOPAL oligomers also occurs in a more PD-relevant cellular environment in which aS is not overexpressed: in primary cortical neurons treated with 20 μM or 50 μM DOPAL and showing synaptic vesicles alterations previously described (Fig. 3c,d). The western blot analysis of aS level and oligomerisation showed that upon DOPAL treatment, monomeric aS was accumulated and aS-DOPAL oligomers were formed in a [DOPAL]-dependent manner. Altogether these results suggest that DOPAL can target both overexpressed aS and endogenous aS, and it is able to induce aS oligomerization in immortalized cell lines, and in primary neurons. The presence of aS-DOPAL oligomers is likely associated with the impairment of homeostasis in synaptic vesicles observed in these models.

### DOPAL modifies aS lysine residues both in cell models and *in vitro*

To characterize the molecular level chemical modifications to aS induced by DOPAL in cell models, we set out to perform Liquid Chromatography-Mass Spectroscopy (LC-MS) experiments. We used HEK293T cells overexpressing aS-EGFP, and treated with 100 μM DOPAL because in these conditions a higher yield in term of DOPAL-modified aS after ABPA resin pull-down was obtained.

We subsequently cut the band at 50 kDa as visualized by silver staining (i.e. where aS-EGFP is detected by western blot) in the APBA resin pull-down SDS-PAGE, and then digested the content of the band with pepsin. The resulting peptides were subjected to LC-MS analysis that showed that K6, K10, K12, K23, K32, K34, K43, K58, K60, K96 and K97 were modified by DOPAL (Table 1).

**Figure 4:**
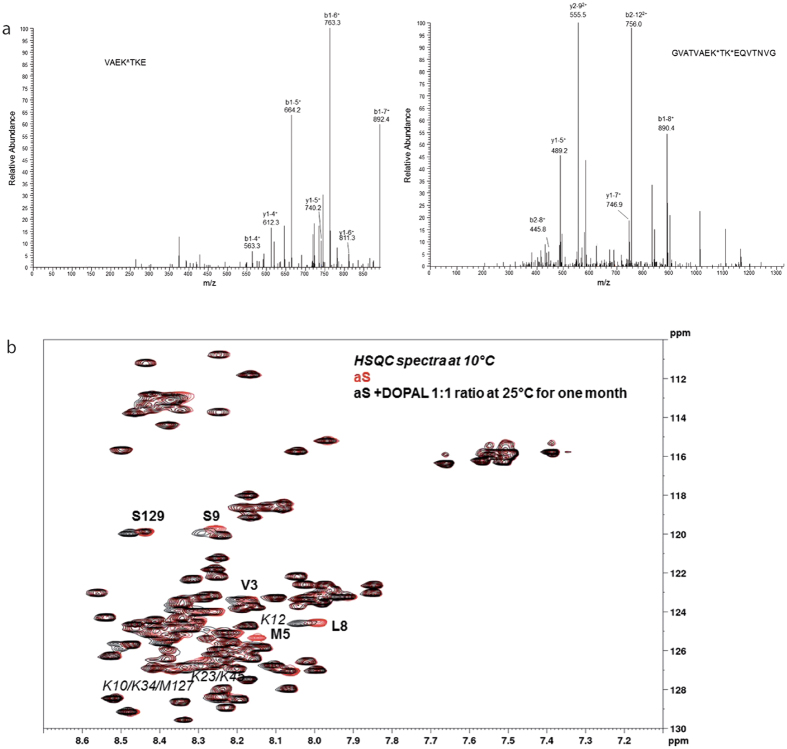
Mass spectra HSQC-NMR spectra of DOPAL modified aS. (**a**) In the left panel, MS/MS spectrum of the peak assigned to the VAEKTKE peptide (55–61 residues of aS) with the K58 residue bound to DOPAL, resulting in a 136 amu increase in peptide mass. This peptide was observed when aS reaction with DOPAL was performed in reducing conditions. The assignments of y and b ion series are reported (all are in mono-charged state). In the right panel, MS/MS spectrum of the peak assigned to the GVATVAEKTKEQVTNVG peptide (51–67 residues of aS) with the K58 and K60 residues bound to DOPAL, resulting in a 134 amu increase in peptide mass for each modification. This peptide was observed in aS-DOPAL in cell cultures experiments. The assignments of y and b ion series are reported (either in mono- or double-charged state). (**b**) Spectra overlap of ^15^N-^1^H HSQC spectra recorded at 10 °C for a sample of ^15^N labelled aS (160.5 μM) alone (red) or incubated with DOPAL for 1 month at 25 °C in a stoichiometric ratio of 1:1 (black).

In parallel, we assessed the DOPAL-induced chemical modification to aS *in vitro*. To this end we incubated recombinant aS and DOPAL in a stoichiometric ratio of 1:20 *in vitro* for 5 h with and without the reducing agent NaBH_3_CN, to mimic a possible contribution of reducing agents in the cell cytoplasm. Then the aS-DOPAL reaction mixture was digested by pepsin and the resulting peptides were separated and analysed by LC-MS. In both non-reducing and reducing conditions, several possible modifications were considered, assuming that DOPAL could react with Lys residues of aS either through the catechol ring or the aldehyde group, as also proposed by others^31^,^35^. The results are summarized in Table 1 and compared to those obtained in cells and from other *in vitro* studies^31^. From these data we can infer that the reaction involving the aldehyde carbonyl is preferred, as in all experiments a mass increase compatible with the addition of a DOPAL-quinone through the aromatic ring (+148 amu on Lys 102) was observed only once. As expected, the presence of NaBH_3_CN changes the redox state of DOPAL adducts; in this condition, a 136 amu mass increase of lysine residues 6, 43, 58, 60 and 80 was observed (Fig. 4a). Furthermore, NaBH_3_CN seems to govern which Lys residues are more likely to be covalently modified by DOPAL. When aS and DOPAL were allowed to react without any reducing agent, a 134 amu mass increase was identified for Lys in positions 32 and 34. An increase of +270 amu was observed for Lys residue in position 80, corresponding to a possible dimer of DOPAL.

In addition to covalent adducts with Lys residues, DOPAL is able to induce methionine oxidation to methionine sulfoxide. Oxidation of Met in positions 1, 5 and 127 was observed in the experiments without NaBH_3_CN, while Met 127 was oxidized even in the presence of NaBH_3_CN. This result is only partially coherent with a recently published characterization of aS-DOPAL oligomers in which Follmer and colleagues found that the aS Lys modified by DOPAL are those at the N-terminus and in the NAC region of the protein, i.e. K10, K12, K21, K23, K32, K34 and K43 (Table 1)^31^.

HSQC-NMR measurements were then performed to follow the kinetics of the process, in an attempt to define the relative reactivity of the individual Lys residues in aS sequence. Given the high reactivity of DOPAL toward Lys^27^, the stoichiometric ratio was set between the protein and the aldehyde to 1:1 to slow down the reaction and improve time resolution. The resulting spectra are reported in Fig. 4b. Only some of the lysine residues modified by DOPAL seem to be involved in the reaction when DOPAL concentration is comparable to aS concentration. In particular, K12, one between K23 and K45, and one among K10, K34 and M127 (in the latter cases the peaks are too close to be discriminated) are the modified residues as detected by HSQC. Interestingly, HSQC also showed that V3, L8, S9 and S129 residues are affected by the reaction. For the first three, the peak shift may be due to the local changes induced by M5 oxidation and K10/K12 modification by DOPAL, while M127 oxidation may be responsible for the shift of the S129 peak.

### DOPAL reaction with aS leads to the formation of aS oligomers *in vitro*

**Figure 5:**
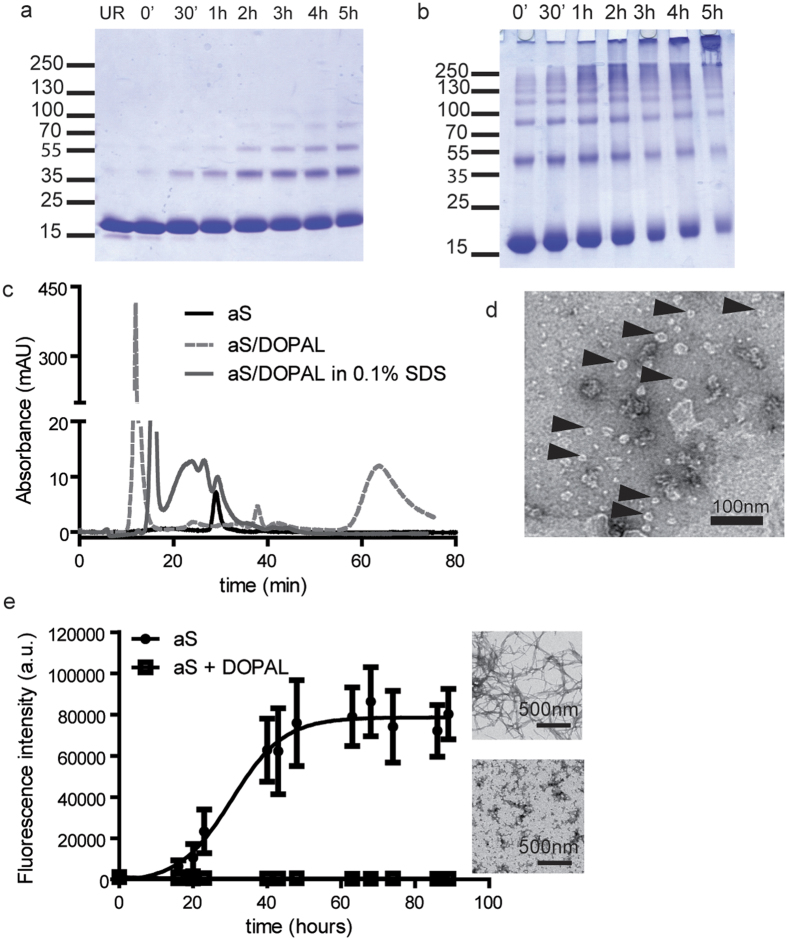
Biophysical and biochemical characterization of aS-DOPAL monomer and oligomers. (**a**) SDS-PAGE of samples from the aS and DOPAL reaction performed at 25 °C for 5 hours collected at different time points. The reaction products were obtained reacting 67 μM aS in a stoichiometric ratio of 1:20 with DOPAL. (**b**) SDS-PAGE showing the reaction products of 310 μM aS in a stoichiometric ratio of 1:5 with DOPAL. (**c**) Size-exclusion chromatography of monomeric aS compared to the reaction products of 67 μM aS in a stoichiometric ratio of 1:20 with DOPAL, which are large oligomers eluting in the void volume of the column. The graph also shows the chromatographic profile of aS-DOPAL oligomers in the presence of 0.1% SDS. The elution profile of aS-DOPAL oligomers changes in the presence of SDS and shows peaks at lower molecular weights further confirming that SDS can disaggregate the aS-DOPAL non-covalent oligomers into the ensemble of dimers, trimers and tetramers reported in panel a. (**d**) TEM image of aS-DOPAL oligomers showing annular shapes. (**e**) ThT kinetic of aS aggregation alone (full circles) shows the typical sigmoidal shape and lead to the formation of canonical amyloid fibrils. The aggregation process of aS-DOPAL molecules (empty squares) shows that they do not acquire a beta-sheet structure and the final products of the aggregation are not fibrils but amorphous aggregates.

With the aim to define the *in vitro* structural and functional properties of the reaction products obtained from aS and DOPAL, and to define the possible mechanism(s) through which aS-DOPAL oligomers alter synaptic vesicles homeostasis, aS and DOPAL were reacted *in vitro*. 67 μM recombinant purified aS was incubated with DOPAL *in vitro* for 5 h at pH 7.4 and 25 °C with a stoichiometric ratio of 1:20. Aliquots of the reaction mixture were collected at different time points (0, 30 min, 1, 2, 3, 4 and 5 h) and loaded onto a SDS-PAGE (Fig. 5a). The reaction produced a time-dependent intensity increase of the bands corresponding to oligomeric forms of aS. By increasing aS concentration to 300 μM while maintaining a 1:20 aS:DOPAL stoichiometry, part of the aS-DOPAL reaction products were trapped in the SDS-page loading wells, being insoluble high molecular weight aggregates (Fig. 5b).

### aS-DOPAL covalent oligomers form non-covalent aggregates in solution

An attempt was made to separate the different oligomeric forms of aS observed by electrophoresis by SEC (Fig. 5c). The elution profile of aS-DOPAL mixture in PBS pH 7.4 after 5 h reaction shows a high intensity peak eluting with the void volume of the column and a higher baseline, instead of the distribution of peaks corresponding to various aS oligomeric species. No peak at the retention time of about 25 min corresponding to monomeric aS was present. When SDS was added to the elution buffer, the peak eluting with the void volume of the column was reduced and broad peaks appeared at shorter retention time compared to monomeric aS. These results suggest that large peak in the void volume is constituted by non-covalent aS-DOPAL large oligomers, which are likely disassembled by the SDS present in the elution buffer. The non-covalent aS-DOPAL oligomers disassembly reveals the presence of smaller covalent aS-DOPAL oligomers that are shown as a ladder in the SDS-PAGE and in broad peaks with a retention time between 17.5 and 25 minutes when performing SEC. The high absorption due to the bound DOPAL hinders the possibility of using the chromatographic profile to quantify the relative amounts of aS, DOPAL and aS-DOPAL adducts at the end of the reaction.

To define the morphology of the aS-DOPAL large oligomers, imaging experiments were performed on the elution peak collected at the void volume of the column. TEM imaging on the products of aS-DOPAL reaction at 5 h confirmed that the oligomeric species are a heterogeneous ensemble mainly constituted by oligomers, some of which have a characteristic annular structure that was already observed for other aS oligomers^36^,^37^,^38^,^39^ (Fig. 5d).

### DOPAL-modified aS cannot form amyloid fibrils

The time evolution of the products of the aS-DOPAL reaction was analysed by Thioflavin T (ThT) aggregation assay using unreacted aS as positive control. The kinetic analysis of the aggregation behaviour of aS control, as measured by the increase of ThT fluorescence intensity over time, showed a typical sigmoidal curve^40^. Conversely, when aS-DOPAL molecules were subjected to the same aggregation protocol, no increase in the ThT fluorescence signal was observed. This result suggests that DOPAL modifications of aS lead to the formation of DOPAL modified aS monomers and aS-DOPAL oligomers that do not convert into beta-sheet aS amyloid fibrils (Fig. 5e). The final products of the aggregation of aS-DOPAL molecules resemble amorphous aggregates rather than the aS fibrils morphology obtained by aggregating monomeric aS alone when analysed by TEM. These results are in agreement with what was published by Follmer *et al*.^31^.

### DOPAL-modified aS monomers and oligomers interact with and permeabilise cholesterol containing lipid membranes

Synaptic vesicles are membranous structures that may be easily damaged by oligomers having the propensity to permeabilise lipid membranes. Given (i) the annular shape of the aS-DOPAL oligomers observed by TEM (Fig. 5d) similar to those able to permeabilise lipid membranes^41^ and (ii) the damages observed in synaptic vesicles in the experiments reported earlier in this manuscript, we investigated the ability of aS-DOPAL oligomers to permeabilise lipid membranes *in vitro*.

**Figure 6:**
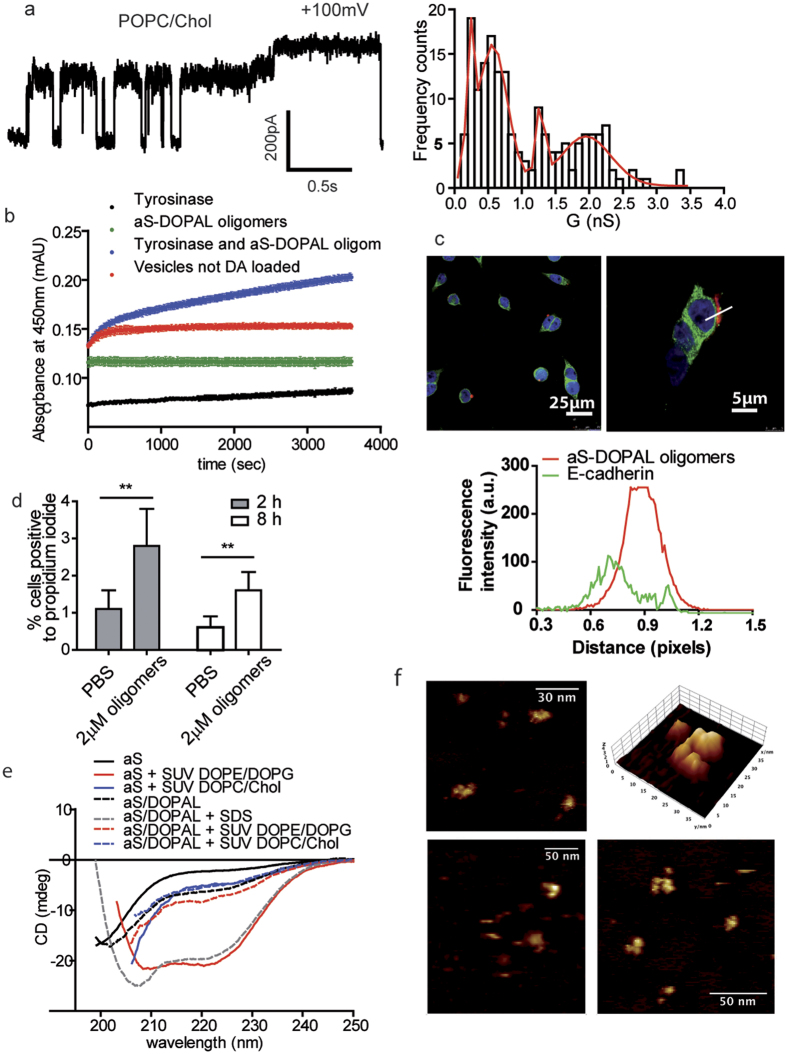
Permeabilization ability of aS-DOPAL oligomers in different membrane models. (**a**) Planar Lipid Membrane experiments showing the permeabilization ability of aS-DOPAL oligomers in lipid membranes constituted by POPC/CHO, and relative conductances distribution to characterize the pores formed. The same permeabilization was not present when the planar membranes were prepared with DOPE/DOPG. (**b**) POPC/CHO SUVs mimicking synaptic vesicles composition and loaded with DA were permeabilized by aS-DOPAL oligomers and allowed the release of DA as measured by monitoring the Tyrosinase dependent Dopachrome formation at 505 nm (blue). When SUVs were not loaded with DA (red) or Tyrosinase was removed from the SUVs solution in the presence of aS-DOPAL oligomers (green), there was no formation of Dopachrome. When only Tyrosinase was added to the DA loaded SUVs (black), a small increase in the measured absorbance was observed probably because of DA leaking. (**c**) Confocal images of aS-DOPAL oligomers (red) localized at the plasma membrane (green) of BE(2)-M17 cells. (**d**) Permeabilization of BE(2)-M17 cells by aS-DOPAL oligomers as measured by propidium iodide after 2 and 8 hours treatment. (**e**) CD measurement of monomeric aS and aS-DOPAL oligomers alone and in the presence or absence of POPC/CHO or DOPE/DOPG SUVs show that aS-DOPAL oligomers can acquire an alpha-helical structure only in the presence of SDS which is able to disaggregate large non-covalent oligomers. (**f**) AFM images of aS-DOPAL oligomers performed on POPC/CHO lipid monolayer in liquid show that they present the annular shape previously observed by TEM by us and for other aS oligomeric species by others.

Since it was previously shown that monomeric aS is more active on negatively charged membranes composed by DOPE:DOPG = 1:1 42, we first performed experiments on planar lipid membranes (PLM) with this lipid composition, to test the permeabilising ability of aS-DOPAL oligomers. However, aS-DOPAL oligomers were unable to affect the stability of these membranes, and no changes in membrane permeability were observed at applied voltages ranging between −150 mV and +150 mV. The results of the PLM experiments radically changed when cholesterol was introduced (POPC:CHO = 1:1) (Fig. 6a). Currents were measured upon ±40 V voltage applications and four levels of conductance were observed and analysed by fitting the conductance distribution (in nS) with a multi-Gaussian profile: c_1_ = 0.23 ± 0.02 nS; c_2_ = 0.57 ± 0.02 nS; c_3_ = 1.28 ± 0.02 nS and c_4_ = 1.9 ± 0.7 nS (R = 0.95) (Fig. 6b). This suggests that a heterogeneous population of oligomers may be responsible for the observed permeabilisation that occurs in cholesterol-containing membranes.

To further investigate aS-DOPAL oligomers permeabilising ability, we performed a calcein release assay (Fig. S1). Incubation of aS-DOPAL oligomers at different concentration (from 0.1 μM to 20 μM) with calcein-loaded POPC:CHO 1:1 liposomes (of an average diameter of 100 nm) did not lead to the expected increase in fluorescence intensity over time when calcein is released. To investigate whether the absence of calcein release was due to the pore selectivity, the absence of an applied voltage, or because the aS-DOPAL oligomers based pores were too small to allow the release of calcein from liposomes, a smaller and more relevant reporter molecule, DA, was chosen. An *in vitro* model for the study of DA release from liposomes mimicking synaptic vesicles was conceived: the hypothesis was that DA, which is smaller than calcein (153.2 *vs.* 622.6 g/mol), could leak out from the pores formed by aS-DOPAL oligomers. If proved, this permeabilisation effect could be a highly specific toxicity mechanism to be ascribed to aS-DOPAL oligomers, i.e. the damage of DA synaptic vesicles in dopaminergic neurons.

To detect DA leak from POPC:CHO = 1:1 vesicles, they were first loaded with DA in phosphate buffer pH 5.6 to minimize DA autoxidation. Then, vesicles were resuspended in solution containing the DA oxidizing enzyme tyrosinase, and the DA-release from vesicles was monitored over time as Dopachrome formation (detected at 450 nm) by tyrosinase. The results showed that the presence of 20 μM aS-DOPAL oligomers induces a significant release of DA compared to the controls (Fig. 6b).

The permeabilisation properties of aS-DOPAL oligomers were then analysed in a cellular model. To test whether aS-DOPAL oligomers would localize at membranes in cells, BE(2)-M17 cells were treated with 2 μM oligomers and their colocalisation with the plasma membrane marker E-cadherin was verified (Fig. 6c). Next, the permeabilisation of BE(2)-M17 cells plasma membrane (due to the treatment with aS-DOPAL oligomers) was measured, using propidium iodide as a marker (Fig. 6d). The obtained results suggest that oligomers maintain their ability to permeabilise the plasma membrane, which has a composition similar to that of synaptic vesicles, providing further support to the proposed role of as-DOPAL oligomers in damaging vesicles.

### Structural and morphological characteristics of aS/DOPAL oligomers when localized at cholesterol lipid membranes

A relevant issue previously discussed in the literature^31^ concerned the ability of the aS-DOPAL oligomers to acquire a specific secondary structure. To address this question, circular dichroism (CD) experiments were performed on aS-DOPAL oligomers in the presence of SDS or liposomes. The results showed that aS-DOPAL oligomers in the presence of SDS acquire an alpha-helical structure very similar to the secondary structure of monomeric aS in the same conditions (Fig. 6e). By measuring CD spectra of aS-DOPAL oligomers in the presence of SUVs, a very different result was obtained. In this latter case, the non-covalent interactions holding together aS-DOPAL covalent oligomers were maintained, and no variations in the CD signal of aS-DOPAL oligomers were observed. These results are in agreement with what was previously shown by Follmer *et al*.^31^ for the measurement performed in the presence of SDS, whereas aS-DOPAL molecules do not show any secondary structure in solution with SUVs in our experiment. This difference may be ascribed to the different lipid composition of SUVs and to the fact that Follmer measured aS-DOPAL monomer, while this study examined aS-DOPAL oligomeric ensemble of species.

Given the PLM and DA release results suggesting that aS-DOPAL oligomers interact with cholesterol containing lipid membranes *in vitro*, the morphology of these oligomers upon deposition on 50% DOPC-50% CHO lipid membranes was observed by atomic force microscopy (AFM). An example of the images obtained is shown in Fig. 6f, in which a 3D representation of aS-DOPAL oligomer is also reported. The observed structure is in agreement with the results obtained in TEM experiments, and further supports that aS-DOPAL oligomers retain their pore-like structure upon interaction with lipid membranes.

## Discussion

This study analysed how the DA catabolite DOPAL can covalently modify aS Lys, both *in vitro* and in neuronal models, leading to the formation of oligomeric species of aS which impair synaptic vesicles homeostasis. Two different strategies were used to study synaptic vesicles functionality in neuronal cells and in primary neurons in the presence of aS-DOPAL oligomers formed upon DOPAL treatment. The first relied on the overexpression of the fluorescent protein synapto-pHluorin targeted to the inside of synaptic vesicles in BE(2)-M17. The results suggest that the aS-DOPAL oligomers permeabilise the synaptic vesicles, and cause protons to leak out and raise the pH within the vesicles. At the same time, vesicle mobility was significantly affected. The second approach was based on TEM quantification of synaptic vesicles in primary neurons treated with DOPAL, in which the endogenous aS is sufficient to generate detectable aS-DOPAL oligomers. The result showed that the oligomers can affect the distribution of synaptic vesicles among the different pools. Furthermore, there is a strong decrease in the number of vesicles per synapse in the neurons treated with DOPAL, a reduction in the fraction of synaptic vesicles in the ready-releasable pool and an increase in the resting pool^34^. These observations, together with the fact that TIRFM detects vesicles at <150 nm from the plasma membrane, suggest that aS-DOPAL oligomers may preferentially damage the vesicles ready to be released at the synapse, possibly because oligomer formation occurs in the subcellular region where aS is known to participate in synaptic vesicle recycling. It should be mentioned that the chemical modifications of aS by DOPAL may also hinder SNARE complex assembly^18^, a proposed physiological function of aS. Therefore, reducing the amount of functional aS at the synapse may affect the number of synaptic vesicles belonging to the different pools and their recycling.

The proposed mechanism by which aS-DOPAL oligomers affect synaptic vesicles, implies their permeabilisation. This permeabilisation process was investigated *in vitro* to analyse all the processes involved including: Lys modification by DOPAL, oligomers formation, oligomers-membrane interaction in relation to membrane composition and selectivity of the released species. Interestingly, aS-DOPAL oligomers share some structural characteristics with other aS oligomers formed *in vitro*^36^,^37^,^38^, as measured by TEM and AFM. The more detailed analysis was obtained from image reconstruction of cryoTEM images and it clearly outlines a pore like structure^39^. Here, in addition to the TEM images that are coherent with previously reported morphologies, we directly observed a pore-like structure by AFM upon deposition of aS-DOPAL oligomers on cholesterol-containing lipid membranes. These structural hints lead to the hypothesis that aS-DOPAL oligomers could be able to permeabilise artificial membranes, as previously suggested for other aS oligomer by this research group^43^ and others^41^. The aS-DOPAL oligomers pore-forming ability was proved in a PLM experiments and in an *in vitro* model for synaptic vesicles. Interestingly, it was observed that aS-DOPAL oligomers permeabilization occurs only when the membrane contained cholesterol, and the same pores did not form in the presence of unmodified monomeric aS, which was shown to mainly bind and permeabilise negatively charged lipid membrane^42^. The observed requirement of cholesterol, recently substantiated for other aS oligomers^44^, and the fact that the membranes used were uncharged, suggest that these oligomers may be more prone to damage membranes with composition similar to those of synaptic vesicles^45^. The aS-DOPAL oligomers were also shown to co-localize at plasma membrane when used to treat BE(2)-M17 cells, hinting again at the binding of the oligomers to physiological membranes. The treatment of BE(2)-M17 neuronal cells with oligomers lead to cell permeabilisation, suggesting that the effect observed *in vitro* also occurs on live cells.

An important piece of information which emerged from the *in vitro* permeabilisation experiments is that DA can easily permeate out of vesicles in the presence of aS-DOPAL oligomers. This implies a self-amplifying mechanism, in which a few molecules of cytoplasmic DOPAL in the presence of aS generate oligomers. These in turn trigger the release of further DA, which at the neutral cytoplasmic pH could be either oxidized or converted to DOPAL, which will generate further aS-DOPAL oligomers. Given that DA neurons, wherein DOPAL may accumulate, are the neurons mainly affected in PD, these aS-DOPAL oligomers could in part account for the specific death of this class of neurons in PD. Interestingly, also other endogenous aldehydes were shown to covalently modify aS (recently reviewed in Plotegher & Bubacco^27^) and form oligomers that may be toxic, although the details of the mechanisms of toxicity are scarce. To make things worse, cytosolic ALDH, a detoxifying enzyme, has been reported to be downregulated in brains of PD patients^7^ and the DA neuronal population lacking the enzyme has been reported to be particularly vulnerable to neurodegeneration and aS aggregation^8^. Besides aS-DOPAL oligomers formation, aS Lys modification itself can be responsible for toxicity mechanisms in neurons. aS Lys residues are essential for the capacity of aS to bind to lipid membranes^46^,^47^ and their modification may cause an increase in the cytoplasmatic fraction of aS. A second direct consequence of aS Lys modification is a decreased capacity for aS to be ubiquitinated, SUMOylated, and acetylated, with the potential to impair the complex signalling pathways associated to these modifications^27^. In this context, the data obtained *in vitro* by MS and HSQC identify several target Lys residues, likely leading to more than one toxicity mechanism. The discrepancy between the data previously published by Follmer *et al*.^31^ and the results presented here in term of aS Lys modifications *in vitro* are likely due to the differences in the preparation of the sample. On the contrary, the differences among aS Lys found to be modified in cells and those modified *in vitro* may be due to the different conformation of aS monomers in cells, which may affect DOPAL reactivity toward aS Lys^26^. Another possibility is that the difference is due to the contribution of reducing species present in the cytoplasm and to other cellular mechanisms that clear DOPAL-modified aS^22^. Moreover, only monomeric aS modified by DOPAL (band at about 50 kDa) was collected from cells, and it seems likely that the digestion of monomeric aS modified by DOPAL may lead to the detection of more modified residues, because of an increased efficiency in the pepsin activity compared to the digestion of aS-DOPAL oligomers. This may also explain why there are more similarities between the modified Lys found in the aS-DOPAL monomers derived from cells and those found in the analysis of aS-DOPAL monomers obtained *in vitro* by Follmer *et al*.^31^. To conclude, the presence of aS-DOPAL oligomeric permeabilising species reported here have to be verified in PD patients, then further studies should focus on understanding how to prevent the formation of aS-DOPAL oligomers, for example by enhancing ALDH activity or expression level, by developing an immunotherapy against aS-DOPAL oligomers, or by acting on the cellular conditions.

## Methods

### Recombinant alpha-synuclein purification

Human aS cDNA was subcloned into the pET28a plasmid (Novagen) as previously described^48^.

Protein was then expressed in *E. coli* BL21(DE3) strain and aS was recovered from the periplasm by osmotic shock. The sample was then boiled for 10 minutes and the soluble fraction, containing the protein, was subjected to an ammonium sulfate precipitation (two step, respectively 35% and 55% of saturation). The pellet was then resuspended, dialyzed against deionized water, loaded into a 6 ml Resource Q column (Amersham Biosciences) and eluted with a 0–500 mM linear gradient of NaCl. Fractions corresponding to aS were collected, dialyzed against deionized water and lyophilized.

### Synthesis of DOPAL

DOPAL was synthesized by rearrangement of epinephrine, following the method by Robbins^49^. The product was characterized by means of NMR spectroscopy using a Bruker AVANCE 400 spectrometer. The purity of obtained DOPAL was confirmed through HPLC by using a Jasco MD-1510 instrument with spectrophotometric diode array detection equipped with a Waters Symmetry C18 reverse-phase column (250 × 4.6 mm). Elution was carried out starting with 0.1% trifluoroacetic acid in water for 5 min, followed by a linear gradient, in 40 min, to 100% acetonitrile with 0.1% trifluoroacetic acid. The flow rate was 0.8 mL/min.

### *In vitro* modifications of alpha-synuclein by DOPAL

Samples of modified aS were prepared by adding 1.25 mM DOPAL to 67 or 300 μM aS solution in 50 mM phosphate buffer, pH 7.4. Two different set of samples have been prepared: with and without NaBH_3_CN as a reducing agent. In both cases reaction mixtures were allowed to react at 37 °C for 5 hours, protected from light. Samples aliquots were collected at different time points, loaded onto a gradient 4–20% SDS-PAGE and stained with Comassie blue. When the reaction was carried out in order to perform further experiments, the final reaction product was extensively dialyzed against PBS to eliminate DOPAL non bound to aS.

### Gel filtration analysis

Separation of different species of aS after DOPAL reaction was obtained by gel filtration using a Superdex^TM^ 200 10/30 GL column (GE Healthcare) connected to an ÄKTAprime plus (GE Healthcare) system. The column was equilibrated with PBS pH 7.4 and the flow rate was 0.5 ml/min.

### TEM imaging on alpha-synuclein/DOPAL oligomers and alpha-synuclein fibrils

15 μl aliquots of the aS/DOPAL oligomers, obtained as previously described, or of aS fibrils were absorbed onto carbon-coated copper grid and a 0.05% uranyl acetate solution was used for negative staining. TEM pictures were taken on a Tecnai G2 12 Twin instrument (FEI Company, Hillsboro, OR, USA).

### LC-MS/MS analysis of protein fragments

After 5 hours HCl was added to reach pH 1.3 and the modified aS was digested at 37 °C with pepsin. The amount of pepsin added was 2% (w/w) with respect to aS and the enzyme was allowed to react overnight. The same amount of pepsin was added again and the digestions were carried out for other 6 hours, before injecting in LC-MS/MS.

aS-EGFP protein was extracted from SDS-page gels after loading the cell lysate and running the gel. A band in correspondence of the molecular weight of the aS-EGFP protein was identified and cut. Pepsin digestion followed as described above. Peptide fragments obtained by digestion of the different aS samples were separated and analyzed in automated LC-MS/MS mode using a LCQ ADV MAX ion trap mass spectrometer coupled with an automatic injector system Surveyor HPLC (Thermo Finnigan, San José, CA, USA) with a BioBasicTM C18 (5 μm, 150 × 2.1 mm) column. The elution was performed using 0.1% HCOOH in distilled water (solvent A) and 0.1% HCOOH in acetonitrile (solvent B), with a flow rate of 0.2 mL/min; elution started with 98% solvent A for 5 min, followed by a linear gradient from 98% to 55% A in 60 min. The MS/MS spectra obtained by CID were recorded with an isolation width of 2 Th (m/z), the activation amplitude was around 35% of ejection RF amplitude of the instrument. For the analysis of protein fragments, the mass spectrometer was set such that one full MS scan was followed by zoom scan and MS/MS scan on the most intense ion from the MS spectrum. To identify the residues, the acquired MS/MS spectra were automatically searched against protein database for aS or aS-EGFP using the SEQUEST^®^ algorithm incorporated into Bioworks 3.1 software (Thermo Finnigan, San Jose, CA).

### Alpha-synuclein/DOPAL HSQC-NMR

We incubated a sample of 15^N^ labelled aS (160.5 μM) produced as previously described^40^ in the presence of an equimolar amount of DOPAL (160.5 μM) in PBS (pH 7.4) at room temperature for 30 days. The reaction was monitored by recording 15N-1H HSQC spectra of the mixture at 25 °C. The final spectra reported are the overlap of the 15N-1H HSQC spectra of aS sample in the absence of DOPAL and after 30 days of incubation with DOPAL at 10 °C. The spectra were recorded at lower temperature than the incubation temperature due to resolution issues. The NMR experiments were recorded with a Bruker FT NMR Avance III 600-MHz spectrometer equipped with a 5-mm CryoProbe QCI 1 H/19F-13C/15N-D quadruple resonance, and a shielded z-gradient coil. The analysis of the spectra was performed using the CcpNMR software and the NMR assignment published in the BMRB (bmr16300.str) for the intrinsically unfolded aS.

### Thioflavin T alpha-synuclein aggregation assay

Fibrillation assay for aS was set up in a 96-well black plate (Perkin Elmer) with flat black bottom. For the control samples, lyophilized protein was dissolved in PBS (10 mM sodium phosphate, 1.8 mM potassium phosphate pH 7.4 and 137 mM NaCl, 2.7 mM KCl) and passed through a 100 kDa MWCO centrifugal filter unit (Amicon^®^ Ultra, Millipore) to retain unsolved aggregates that can act as seeds. Protein concentration was determined using a calculated extinction coefficient (Abs 0.1%) at 280 nm in water of 0.412 ml/(mg*cm); for the modified protein the concentration was referred to the alpha-synuclein before DOPAL reaction and filtration was avoided because reacted material was retained by the filter. For each protein were prepared 3 wells: each well contains 200 μl of sample, constituted of 1 mg/ml (about 70 μM) protein, 20 μM ThT, 0.05% sodium azide. The plate was sealed with MicroAmp^®^ Optical Adhesive Film (Applied Biosystems^®^ - Life technologies) to avoid evaporation and incubated into a thermostated orbital shaker (Talboys Professional Model 1000MP Microplate Shaker by Troemner) at 37 °C at 1000 rpm. Aggregation progression was monitored measuring ThT emission with a VICTOR™ X3 Multilabel Plate Reader (PerkinElmer) at indicated intervals of time. ThT fluorescence was measured with excitation at 450 nm and emission at 535 nm, with a measurement time of 0.1 s, a CW-lamp energy of 5000 and a small emission aperture.

### Planar Lipid Membranes (PLMs) measurement

Solvent-free PLMs were prepared using equimolar 1,2-dioleylphosphatidyl-glycerol (DOPG) and 1,2-dioleyl-phosphatidyl-ethanolamine (DOPE) or equimolar 1-palmitoyl-2-oleoyl-sn-glycero-3-phosphocholine (POPC) and cholesterol suspended in pentane, as described in ref. 50. All experiments were started in symmetric conditions, using 5 mM Hepes, 100 mM KCl (pH 7.0) buffer and miniature magnetic stirrers to mix the solutions in both chambers. A defined voltage of typically +100 mV was applied across the membrane. The aS/DOPAL oligomers were added to *cis* side in concentration ranging from 0.1 μM to 1 μM. The current across the bilayer was measured and the conductance G was determined using the relationship:


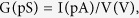


where I is the current across the membrane and V the applied transmembrane potential.

Macroscopic currents were recorded by a patch clamp amplifier (npi, Tamm, Germany), filtered at 100 Hz, digitalized and acquired at 2 kHz by the computer using a DigiData 1322 A/D converter and pClamp software (Axon Instruments, Sunnyvale, CA). All the measurements were performed at 22 °C.

### Preparation of Small Unilamellar Vesicles (SUVs)

A mixture of 50% 1-palmitoyl-2-oleoyl-sn-glycero-3-phosphocholine (POPC) and 50% 1-palmitoyl-2-oleoyl-sn-glycero-3-phosphate (POPA) or cholesterol were used for the preparation of SUVs. The lipids were dissolved in 1 mL of a 4:1 chloroform/methanol mixture, and the solutions were evaporated under vacuum at 4 °C in a glass test tube. The dry lipid film was suspended in 50 mM sodium phosphate buffer (pH 7.4) to give a final concentration of 30 mM and mixed for 1 h above the melting temperature. The product of hydration was filtered through a large pore size (0.45 μm) filter and, subsequently, extruded through a 50 nm pore filter.

When DA-loaded SUVs were needed, DA was suspended in phosphate buffer pH 5.8 to a final concentration of 100 mM. The dry lipid film was then suspended in the DA solution and SUVs extruded as previously described. SUVs were then washed using Sepharose resin to remove the residual DA and change the buffer to 5 mM HEPES 100 mM KCl pH 7.0.

### CD Experiments

CD measurements were carried out on a JASCO J-715 spectropolarimeter interfaced with a personal computer. The CD spectra were acquired and processed using the J-700 program for Windows. All experiments were carried out at room temperature using HELLMA quartz cells with Suprasil windows and an optical path length of 0.1 cm. All spectra were recorded in the wavelength range of 200–250 nm, using a bandwidth of 2 nm and a time constant of 2 s at a scan speed of 50 nm/min. The signal:noise ratio was improved by accumulating 4 scans. Spectra were acquired with a 5 μM solution of WT aSN in the presence of 50 mM phosphate buffer (pH 7.4) with or without 10 mM SDS or 4 mM SUVs. The same measurements were repeated with an amount of DOPAL oligomers supposed to be equivalent in terms of monomeric aS. Spectra are not normalised respect to the protein concentrations, because of difficulty on quantification of oligomeric species.

### Dopamine release from SUVs detected by a Tyrosinase-based *in vitro* assay

To measure DA release from SUVs in the presence of aS/DOPAL oligomers, 50 μl of DA-loaded SUVs were resuspended in 5 mM HEPES 150 mM KCl pH 7.0 with 0.2 μM tyrosinase (specific activity 6450 U/μM) to a final volume of 200 μl and plated in a 96-well plate. Up to 20 μM aS-DOPAL oligomers were added to the vesicles and monomeric aS and PBS buffer were used as controls. SUVs not loaded with DA were also used in the same experimental conditions to exclude that the effect we measured was due to the experimental conditions or to the oligomers themselves. Dopachrome formation was measured at 505 nm with a plate reader (Molecular Devices UVmax Kinetic Microplate Reader).

### AFM on lipid bilayer

Mica, glued to a metal disc, was freshly cleaved to obtain smooth surface. The lipid membranes were prepared by successive apposition of two 50–50% POPC:CHO (or EggPC:CHO) (Avanti Polar Lipids) monolayers on the mica (similarly to ref. 51) Protein was added at two different concentrations, 5 to 20 μg in about 75–100 μl. After 30–60 minutes incubation at RT, samples were carefully washed and analyzed by AFM at 21 °C. An Asylum Research Cypher, equipped with a droplet tip holder was used to acquire AFM data in liquid environment. Olympus TR400 and BL-AC40 cantilevers were used in tapping mode, scanning the sample with a scan rate of ~1 Hz. Asylum provided software was used for data acquisition. Image analysis was performed with SPIP (v4.2.6.0), applying line-by-line height compensation.

### HEK293T and BE(2)-M17 cells culture and transfection

HEK293T cells media was high glucose DMEM (Life Technologies) supplemented with 10% v/v FBS (Life Technologies), 50 U/ml penicillin and 50 mg/ml streptomycin (Life Technologies) and BE(2)-M17 cells media was DMEM/F12 (Life Technologies) with 10% v/v FBS and 50 U/ml penicillin and 50 mg/ml streptomycin. All the cells were cultured at 37 °C in 5% CO_2_. Cells were transfected with the plasmids aS-EGFP, aS-mCherry or their empty vector, respectively pEGFP-N1 and pmCherry-N1(Novagen). HEK293T were transfected with Polyethylenimine (PEI) in a 1:4 ratio with DNA (v/w), BE(2)-M17 with lipofectamine (Lipofectamine 2000, Invitrogen) in a 1:2 ratio with DNA (v/w).

### Immunocytochemistry, propidium iodide and imaging

BE(2)-M17 cells were plated on glass coverslips at 3 × 10^5^ cells/well, treated with 2 μM aS-DOPAL oligomers for 8 hours and fixed with 4% PFA. Cells were then stained with antibodies against aS (BD Biosciences, 610787) and against the plasma membrane marker E-cadherin (Santa Cruz, SC-7870) and further counterstained with secondary antibodies (Thermo Fisher Scientific, Alexa Fluor 568 A11031 and Alexa Fluor 488 A11034, respectively), and Hoechst (Thermo Fisher Scientific) to visualize nuclei. Imaging was performed on Leica SP5 confocal microscope.

To measure cell permeabilization 120000 cells per well were plated in a 12-well plate. After 24 hours cells were treated with 2 μM aS-DOPAL oligomers and then, after 1 hour and 7 hours cells were stained with 2 μg/ml propidium iodide (Termo Fisher Scientific, P3566) and imaged by a widefield inverted microscope (Leica DMI 4000B).

### Synapto-pHluorin confocal and TIRF imaging and analysis

BE(2)-M17 were plated on glass coverslips at 3 × 10^5^ cells/well, co-transfected with 2 μg synapto-pHluorin and 2 μg mCherry or aS-mCherry for 3 hours by means of lipofection (Lipofectamine 2000, Invitrogen) and 24 or 48 hours after plating cells were treated with 100 μM DOPAL in Optimem. Confocal imaging was performed on Leica SP8 to confirm colocalization between mCherry/aS-mCherry and synapto-pHluorin. Cells were fixed in PFA 4% and counterstained with Hoechst (Invitrogen) to visualize nuclei.

Single-cell imaging under TIRF illumination was carried out on live cells at 1 frame per second for 40 seconds in the continuous presence of Krebs (KRH) solution, 125 mM NaCl, 5 mM KCl, 1.2 mM MgSO_4_, 1.2 mM KH_2_PO_4_, 25 mM 4-(2-Hydroxyethyl)piperazine-1-ethanesulfonic acid (HEPES) (buffered to pH 7.4) and 2 mM CaCl_2_ (Sigma Aldrich), at 37 °C with or without DOPAL 100 μM at different time points. Up to eight cells were imaged on each coverslip in three independent experiments by means of an AxiObserver Z1 inverted microscope (Carl Zeiss Inc.) equipped with an Argon laser at 37 °C using a 100 × 1.45 numerical aperture (NA) oil immersion objective. Green fluorescence was excited using the 488-nm laser line and imaged through a band-pass filter (Zeiss) onto a Retiga SRV CCD camera. TIRF images were analyzed using Image-Pro Plus Analyser Image Software (Media Cybernetics, Bethesda, MD, USA). A set of automated image processing macro/subroutines was developed on the basis of existing algorithms of the Image-Pro Plus Analyser software (nearest neighboring deconvolution, High Gaussian filtering). The resulting corrected images were then analyzed for selection and quantification of fluorescent spots according to their shapes, size and intensity. For each recorded cell image, the total number of vesicles, the average vesicle mobility and fluorescence intensity, the ratio between fixed (vesicles visible in at least 30 out of the 40 frames) and mobile vesicles and its associated standard deviation in each frame were calculated.

### Primary neuronal cell cultures and DOPAL treatment

All animal procedures were performed following the guidelines issued by the European Community Council Directive 2010/63/UE and approved by Ethics Committee of the University of Padova (Project ID: 46/2012).

Neuronal cells were derived from postnatal mouse (P0-P1) brains (CD1 strain). Cerebral cortices were isolated and cells mechanically dissociated in EBSS (Sigma Aldrich). Cells were centrifugated, resuspended in Neuro basal media (Life Technologies) supplemented with 2% v/v of B27 supplement (Invitrogen), 0.5 mM L-glutamine (Life Technologies), penicillin (100 Units/ml) streptomycin (100 μg/ml) and 2.5 μg/ml fungizone (Life Technologies). Cells were then plated at 2 × 10^6^ cells/well on poly-L-lysine (0.1 mg/ml, Sigma Aldrich) coated wells of a 6-well plate and cultured at 37 °C in 5% CO_2_. After 7 days half medium was replaced and the neuronal culture was maintained until day 14. At 14 days neuronal cells were treated for 14 hours with 20 μM and 50 μM DOPAL and processed for further experiments.

### Western blotting and ABPA resin

HEK293T, BE(2)-M17 and neuronal cells were collected and lysed on ice in lysis buffer 20 mM Tris-HCl pH 7.5, 150 mM NaCl, 1 mM EDTA, 1% Triton, 2.5 mM Sodium Pyrophosphate, 1 mM beta-glycerophosphate, 1 mM sodium orthovanadate supplemented with protease cocktail inhibitor (P8340, Sigma). Protein concentration was determined by BCA assay (Pierce) and equal amounts of total protein were loaded. For ABPA resin (A8530 Sigma) pulldown, 50 μg total protein were incubated with 50 μl of the resin overnight at 4 °C shaking. The resin was then pelleted, the supernatant removed and the resin was washed 2 times with PBS/acetronitrile (50:50) and finally with water. Protein was collected from the resin by adding 20 μl Laemmli buffer, loaded into 10% or 4–20% gradient SDS-PAGE and compared with the total lysate. aS detection was performed using antibodies against aS (Abcam, ab138501 for human cells and ab52168 for mouse neurons) and housekeeping proteins (beta-tubulin and HSP90) were detected using a loading control sample kit (OriGene, TA150046). Horseradish peroxidase-conjugated anti-mouse IgG or anti-rabbit IgG were then used and immunoreactive proteins were visualized using enhanced chemioluminescence plus (ECL+, GE Healthcare).

### TEM imaging on vesicles and analysis

Control and DOPAL treated neurons were fixed with 2.5% glutaraldehyde in 0.1 M sodium cacodylate buffer pH 7.4 for 1 hour at 4 °C. Neurons were then postfixed with a mixture of 1% osmium tetroxide and 1% potassium ferrocyanide in 0.1 M sodium cacodylate buffer for 1 hour at 4° and incubated overnight in 0.25% uranyl acetate at 4 °C. After three water washes, cells were dehydrated in a graded ethanol series and embedded in an epoxy resin (Sigma-Aldrich). Ultrathin sections (60–70 nm) were obtained with an Ultrotome V (LKB) ultramicrotome, counterstained with uranyl acetate and lead citrate and viewed with a Tecnai G2 (FEI) transmission electron microscope operating at 100 kV. Images were captured with a Veleta (Olympus Soft Imaging System) digital camera. EM images have been processed on NIH ImageJ before performing the analysis on LoClust tool^52^. Single vesicle has been manually annotated and the distance to the active zone is expressed in nm.

### Statistical analysis

Data analysis, frequency count and statistical analysis (ANOVA and t test) were performed using OriginPro 8.0 or GraphPad Prism 5.0.

## Additional Information

**How to cite this article**: Plotegher, N. *et al*. DOPAL derived alpha-synuclein oligomers impair synaptic vesicles physiological function. *Sci. Rep.* **7**, 40699; doi: 10.1038/srep40699 (2017).

**Publisher's note:** Springer Nature remains neutral with regard to jurisdictional claims in published maps and institutional affiliations.

## Supplementary Material

Supplementary Information

## Figures and Tables

**Table 1:** Comparison among aS residues modified by the reaction with DOPAL identified by this group and others using MS and HSQC-NMR.

	Recombinant aS monomer modified by DOPAL – MS (Follmer *et al*.^31^)	Recombinant aS-DOPAL oligomers in reducing conditions (NaBH_3_CN) - MS	Recombinant aS-DOPAL oligomers - MS	DOPAL-modified aS monomers extracted from cells - MS	Recombinant aS-DOPAL reaction – HSQC (Follmer *et al*.^31^)	Recombinant aS-DOPAL reaction – HSQC
M1	+16		+16			
M5	+16		+16		x	x
K6		+136		+134		
K10	+134			+134	x	*
K12	+148/+166/+168			+270	x	x
K21	+148					no
K23				+136	x	**
K32	+134		+134	+134/+136		no
K34	+166/+168		+134	+134		*
K43	+148	+136		+134		
K45						**
K58		+136		+134		no
K60		+136		+134	x	
K80		+136	+270		x	no
K96				+134		no
K97				+136		no
K102					x	no
M116				+16	x	
M127		+16	+16		x	*

*Means one or more among K10/K34/M127. **Means one or both between K23/K45.
