# Supplementary material for: DOPAL derived alpha-synuclein oligomers impair synaptic vesicles physiological function
Source: Sci Rep. 2017 Jan 13;7:40699. doi: 10.1038/srep40699 (PMC5233976; doi:10.1038/srep40699)
Supplement: Supplementary Information [file srep40699-s1.pdf]

DOPAL derived alpha-synuclein oligomers impair synaptic vesicles physiological function

N. Plotegher[1,2], G. Berti[1], E. Ferrari[3,4], I. Tessari[1], M. Zanetti[5,6], L. Lunelli[5,6], E. Greggio[1], M. Bisaglia[1], M. Veronesi[7], S. Girotto[7], M. Dalla Serra[5], C. Perego[8], L. Casella[3], L. Bubacco[1*]

* Correspondence: luigi.bubacco@unipd.it

1. Department of Biology, University of Padova, Italy
2. Curr. address: Department of Developmental and Cell Biology, University College London, UK
3. Department of Chemistry, University of Pavia, Italy
4. Institute of Biomedical Technologies, National Research Council of Italy, Segrate (Milan), Italy.
5. Institute of Biophysics, National Research Council of Italy, Trento, Italy
6. Laboratory of Biomolecular Sequence and Structure Analysis for Health, BKF, Trento, Italy
7. Department of Drug Discovery and Development, Istituto Italiano di Tecnologia, Genova, Italy
8. Department of Pharmacological and Biomolecular Sciences, University of Milan, Italy

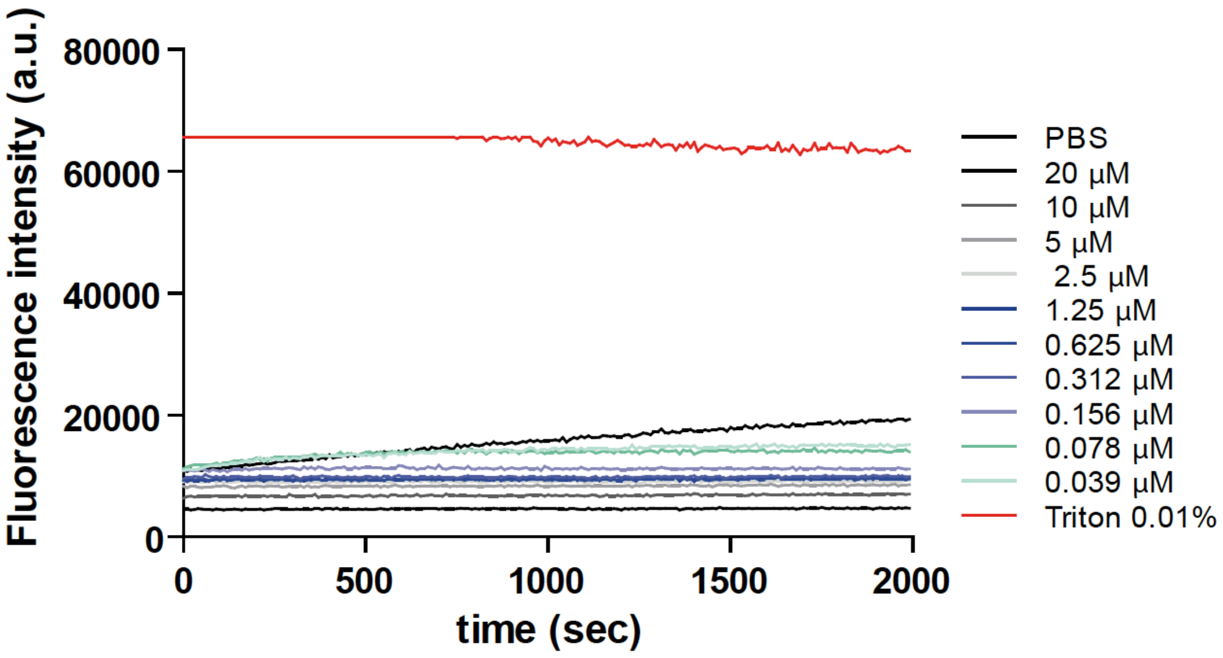

Supplementary Figure S1. aS-DOPAL oligomers do not cause calcein release from SUVs

POPC/CHO SUVs mimicking synaptic vesicles composition and loaded with calcein were incubated with aS-DOPAL oligomers at different concentration and calcein fluorescence was measured over time to detect calcein release induced by permeabilization. None of the aS-DOPAL oligomers concentration tested showed any calcein release. This result suggests that the pores formed by aS-DOPAL oligomers as measured by PLM experiments may need voltage application or that their diameter is too small to allow calcein release.
